# Splice variants of RAS—translational significance

**DOI:** 10.1007/s10555-020-09920-8

**Published:** 2020-08-08

**Authors:** Erzsébet Rásó

**Affiliations:** 2nd Department of Pathology, Semmelweis University, Budapest, Hungary

**Keywords:** HRAS, KRAS, NRAS, Splicing, Expression, Function

## Abstract

One of the mechanisms potentially explaining the discrepancy between the number of human genes and the functional complexity of organisms is generating alternative splice variants, an attribute of the vast majority of multi-exon genes. Members of the RAS family, such as NRAS, KRAS and HRAS, all of which are of significant importance in cancer biology, are no exception. The structural and functional differences of these splice variants, particularly if they contain the canonical (and therefore routinely targeted for diagnostic purposes) *hot spot* mutations, pose a significant challenge for targeted therapies. We must therefore consider whether these alternative splice variants constitute a minor component as originally thought and how therapies targeting the canonical isoforms affect these alternative splice variants and their overall functions.

## Alternative splicing—it is the rule, not the exception

Alternative splicing (AS) allows for two or more mRNA variants to be transcribed from a single gene, potentially coding structurally and functionally different proteins, sometimes with opposing biological functions [1]. Almost all human multi-exon genes have alternative splice variants [2, 3] allowing for an average of 6.3 alternatively spliced transcripts per each of the 20,687 protein-coding genes according to the data from the Encyclopedia of DNA Elements (ENCODE) Consortium. The alternative splice pattern of any given gene is time and space dependent, i.e. differs depending on tissue type and developmental stage even under physiological conditions [4]. The TISA (tissue-specific alternative splicing) project predicted 12,711 genes, 16,016 transcripts and 1035 AS events to be tissue-specific [5] using the Cancer Genome Anatomy Project (CGAP). In case of cancers, an alternative splice pattern can be different from that of the tissue of origin and can be the ‘natural’ result of the altered tumoural microenvironment initiating the change in AS pattern (ASP). Another causative mechanism might be the mutation or post-translational modification of one of the elements of the otherwise finely regulated alternative splicing machinery. The basic mechanism of alternative splicing is that the aforementioned alternative splicing machinery (i.e. the spliceosome, which consists snRNP and snRNA) identifies intronic and exonic sequences based on the presence of consensus splice sites (donor splice site, the last three nucleotides of the exon and the first six of the intron; acceptor splice site, last twenty nucleotides of an intron and the two first of the next; branch sequence). Unlike the single mRNA generated during constitutive splicing, alternative splice variants can be generated via five different alterations during mRNA editing: cassette exon (CE) or exon skipping, alternative 5′ splice site (A5SS), alternative 3′ splice site (A3SS), mutually exclusive exons (MXE) and retained intron (RI). In tumoural tissue, almost all elements of this complex system can carry a mutation changing the ASP of the tissue of origin, often generating tumour-specific splice variants. If mutation occurs in a gene not participating in the regulation of alternative splicing (e.g. splice site mutation), the change will most likely affect the ASP of the gene itself [6–10]. However, mutations within a splice factor (SF) gene might have an effect on the ASP of several of its target genes. Recurrent somatic mutations in one of the core subunit genes of spliceosomes have been described in the majority of tumour types, although most of these occur in haematological malignancies such as MDS, acute myeloid leukaemia and chronic lymphocytic leukaemia [11]. Over half of MDS and 5–10% of AML patients have underlying mutation of one of the splicing factors, such as S*F3B1*, *SRSF2*, *U2AF, ZRSR2,* LUC7L2, PRPF8 or SF3B1 [12, 13], although it is not uncommon in other tumour types either. Seiler M et al. [14] performed systematic analysis of the whole genomes of 33 tumour types from The Cancer Genome Atlas (TCGA) database and identified function altering (oncogene-like, tumour suppressor-like) mutation in 119 splicing factor genes. Despite the fact that only 1.5% of the 3 billion base pairs of the human genome form protein-coding genes and within these the footprint of the few base pairs of splice sites is not strictly defined as consensus splice site, the proportion can even be smaller, but the frequency of mutation in these areas is disproportionately high [15]. The databases generated from the results of high-throughput transcriptome sequencing (RNA-seq) studies also seem to confirm this [16, 17]. For example, searching for splice-site-creating mutations within the 86,565 tumour sequences recorded in The Cancer Genome Atlas (TCGA) database, Jayasinghe et al. [18] identified 1964 originally mis-annotated mutations clearly resulting in new splice junctions. New tumour-specific isoforms generated this way can and already have become key players in the targeted therapy of tumours [19–22]. Recent splicing array data comparing normal and tumour cells is suggestive of the importance of tumour-specific isoform signature over gene signature [23]. The presence of tumour-specific splice pattern has been described in several tumour types such as hepatocellular carcinoma [24], breast carcinoma [25], lung carcinoma [26], AML [27], neuroblastoma [28] and carcinoma of the digestive tract [29]. It is also important to point out that the appearance of a number of these newly identified alternative splice variants (BCR-Abl35INS, BIM-γ, IK6, p61 BRAF V600E, CD19-∆2, AR-V7 and PIK3CD-S) seems to confer resistance to targeted and/or immunotherapy [30]. Therefore, regardless of which component of the complex alternative splicing system is affected by mutation, new or ectopically appearing ‘old’ variants of a gene might be of diagnostic importance. The neoantigenes generated this way appear to be more immunogenic than the canonical protein coded by the same gene and harbouring a missense point mutation. They might even be developed to novel targets of therapy [31–33].

## KRAS, NRAS, HRAS—canonical isoforms—the ‘p21’ proteins and their mutations

Members of the RAS superfamily are classed into five major branches (RAS, RHO, RAB, RAN and ARF) based on the similarity within their sequences and functions [34]. Most studied are KRAS, NRAS and HRAS, oncogene members of the RAS superfamily. These are plasma membrane-bound low molecular weight GTPases (∼ 21 kD, the source of their original name of ‘p21’ proteins), which cycle between the GDP-bound (inactive) and the GTP-bound (active) state and bind and activate a number of downstream effector proteins, such as Raf kinases, phosphatidylinositol 3-kinases (PI3-K) and RalGDS family members. All three are ubiquitously expressed from yeast through insects to every single cell of mammals highlighting their fundamental biological importance. Their structural and functional conservation is such that they are functional even in a heterologous system [35, 36]. For example, spore formation in *Saccharomyces cerevisiae* KO for both of its RAS genes (ras^1−^, ras^2−^) can be restored by human HRAS gene under the control of human GAL10 promoter. Despite the above, the three isoforms fulfil different functions from ontogenetic, tissue function and tumour biology point of view as well. Early studies showed that KRAS was essential for the embryonic development of mice [37]. The physiological functions, frequency of tumours and lifespan of KRAS^±^ knockout mice were identical to those of wild-type (KRAS^+^/^+^) animals. In contrast, KRAS^−/−^ animals (resulting from crossing heterozygous animals) were not viable as the ventricular myocardium showed significant thinning in a 15.5-day-old embryo due to the inadequate proliferation of myocytes. Additionally, neurological, haematological and liver defects also appeared in these animals. In contrast to K-ras, neither H-ras^−^/^−^ nor N-ras^−^/^−^, in fact not even the double KO (i.e. H-ras^−^/^−^ and N-ras^−^/^−^) genotype has an effect on the embryonic development, growth, fertility or neuronal differentiation of rodents [38]. KRAS expression is not increased in HRAS^−/−^ NRAS^−/−^ animals [39], and inversely, HRAS and NRAS expression are not increased in KRAS^−/−^ animals [38]. At the same time, when the KRAS gene was modified to express HRAS protein, HRAS fulfilled the role KRAS would play during embryogenesis, i.e. the offsprings were born, but not its role in cardiovascular homeostasis as the adult animals show dilatative cardiomyopathy associated with arterial hypertension [40].

**Fig. 1:**
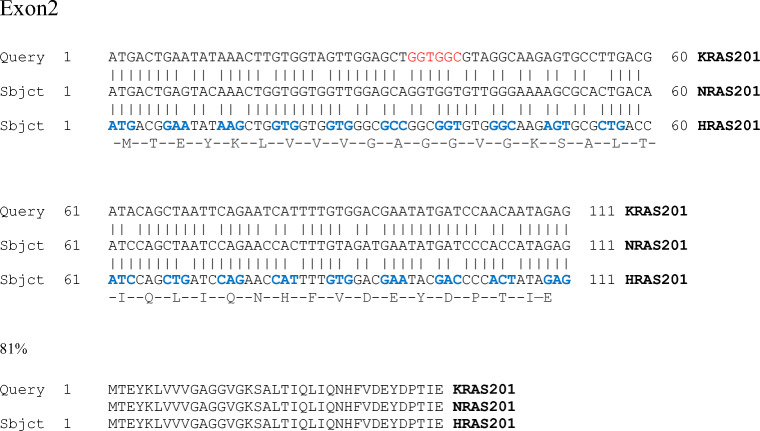
Comparison of the coding nucleotide sequences of exon 2 of the three RAS molecules reveals that even though 57% of the codons are coded by different triplets, protein level homology is 100%

Comparing the structure of the tree isoforms, the effector lobe (1–86aa) situated at the N-terminal region and including the switch I (30–38aa) and switch II (60–76aa) responsible for the molecule’s functional activity shows 100% homology at amino acid level. Similarly, high level, almost 100% homology can be observed in the entire catalytic or G-domain (1–166 aa) itself. The functional differences arise from compartmentalisation and membrane localisation, which are regulated by the hypervariable region (HVR, 167–188/189aa) on the C-terminal. The prerequisite of membrane binding is post-translational modification of the HVR region, and differences in this process will result in differences in membrane localisation. The first step, which is identical in all three isoforms, is farnesylation of the C-terminal cystein by the farnesyl transferase in the endoplasmic reticulum. HRAS and NRAS as well as the KRAS4A alternative splice variant (see detailed description below) proteins are also palmitoylated on additional cysteine residues in the C terminus by RAS palmitoyltransferase. KRAS4B is not palmitoylated; however, for membrane association, it requires a polybasic sequence along with the farnesyl group [41]. It is important to point out that even though the N-terminal regions of KRAS, NRAS and HRAS show 100% homology in amino acid sequence, their coding regions display only 79–82% homology at base sequence level, thoroughly making advantage of the degeneracy of codons. For example, in the case of exon 2, this means that 57% of the codons are encoded by different triplets (Fig. 1).

Therefore, all base variations are permitted from a mutation point of view, as long as they do not affect the conserved amino acid sequence. However, three particular codons are exceptional from this point of view. Codons 12, 13 and 61 are considered to be hotspots; mutation is permitted for all base changes without exception. Activating mutations result in the cessation of the protein’s catalytic activity freezing the oncoprotein into its signal propagating structural conformation and ensuring the survival of tumour cells with unbalanced equilibrium. Essentially it acts as a built in alternative option which the cell can utilise when it ‘gets into trouble’. A number of animal experiments have shown that mutations induced via chemical carcinogenesis within these hotspot codons of the RAS genes will lead to the development of certain tumour types. For example, N-nitroso-N-methylurea (NMU) induces G > A transition in codon 12 of the rat HRAS gene resulting in the development of mammary tumours (NMU-induced rat mammary tumours) [42, 43] and dimethylbenzanthracene (DMBA)-induced A > T transition in codon 61 of mouse HRAS will result in the development of various skin tumours [44]. However, Cha et al. have suggested that chemical carcinogens do not in fact cause direct mutations rather they facilitate the enrichment of the population harbouring the mutation and already present in the normal tissue [45, 46]. They later proved, using a variety of methodologies on a large number of samples, that in self-renewing tissues, over 50% of somatic mutations, including those of the RAS genes, are already present before tumour initiation [47]. The difference between the three isoforms is in the frequency of these hotspot mutations. According to the COSMIC database, of all the coding missense mutations in KRAS, 80% affects codon 12, 14% codon 13 and only 2% codon 61. In NRAS 62% of the missense mutation are in codon 61, 23% in codon 12 and 11% in codon 13. In HRAS this figure is 24% in codon 12, 23% in codon 13 and 40% in codon 61. Mutations affecting other codons can also occur *germ line* and have been shown to be associated with various developmental disorders, such as Noonan, Costello and cardio-faciocutaneous syndromes [48, 49]. However, comprehensive review of available data (COSMIC database) shows that 86–96% of all missense RAS mutations affect codons 12, 13 and 61. This is a remarkable ‘hit rate’ considering that the three genes are in completely different genomic localisation [KRAS on chromosome 12 (12p12.1), NRAS on chromosome 1 (1p13.2) and HRAS on chromosome 11-es (11p15.5)] and the dramatic difference in the number and proportion of bases within the introns (KRAS 50 kbp, NRAS 15 kpb and HRAS 4.5 kbp). Even though they are constitutively expressed proteins, the level of expression can be different across cell types. This propensity is often used to explain the tumour-specific distribution of their mutations [50, 51]. According to the data in The Cancer Genome Atlas (TCGA), KRAS mutations most often occur in endoderm-derived tumours such as pancreatic carcinoma, genomically stable colorectal cancer and lung adenocarcinoma. HRAS mutations most often encountered in thyroid, bladder and kidney carcinomas while NRAS mutations in hepatocellular carcinoma, melanoma and haematological malignancies [52]. KRAS appears to be mutated in 22% of the overall tumour population, while the frequency of HRAS mutation is 2% and NRAS mutation is 8% according to cumulative data [53].

Even though the structure of the isoforms is similar, they are likely to participate in different signal pathways, and the various mutations result in different biological outcomes. Guanine nucleotide exchange factors (GEFs) and GTPase-activating proteins (GAPs) regulate the GTP and GDP exchange on RAS proteins [54]. The effect of extracellular ligand activated cell surface receptors is mediated by guanine nucleotide exchange factors (GEFs, such as Sos 1, Sos 2, Ras-GRF/CDC25^Mm^, Ras-GRF 2 and Ras GRP), which transform the inactive GDP-bound RAS molecule into active GTP-bound form. The reason behind the different behaviour of various RAS isoforms is their variable level of activation by GEFs. For example, Ras-GRF/CDC25^Mm^ activates HRAS but not NRAS or KRAS4B [55]. The picture is further complicated by the fact that certain GEFs are only expressed in very specific tissue manner [56]. GTPase-activating proteins (GAPs) act to return the GTPases to their GDP-bound state. Hence, Ras can in principle be activated by activation or inhibition of relevant GEFs and/or GAPs. Voice JK et al. [57] examined the *in vivo* Raf-1 activating ability of four different RAS isoforms, which are constitutively active due to G12V mutation in Cos-1 cells. The mutant isoforms were transfected into the cells; the measured increase in activity could therefore not have been the result of a change in expression level. According to their results, KRAS4B was 8.4 times more efficient than HRAS, 4.4 than NRAS and 2.3 more efficient than KRAS4A in activating Raf-1. They also assessed the *in vivo* biological effects of these active isoforms by transfecting the G12V-mutated isoforms into three different cell lineages (NIH 3T3 mouse fibroblast, Rat-1 fibroblast and RIE-1 rat intestinal epithelial cell). Examining their focus forming abilities in parallel, they found that HRAS- and KRAS4A-transfected NIH 3T3 and Rat-1 cells showed ∼ 2–2.5-fold, while RIE-1 cells showed ∼ 8.3- and 6.3-fold increase when compared with their KRAS4B- and HRAS-transfected counterparts. The migration propensity of COS-7 cells was significantly increased by KRAS4B, minimally induced by HRAS and was unaffected by KRAS4A and NRAS. Their effect on anchorage-independent growth, which is of significant importance in tumour biology, was summarised as follows: KRAS4A >/= NRAS >>> KRAS4B = HRAS = no growth. We must therefore question whether we can disregard the alternative splice variants of the three RAS genes, as they are significantly different both structurally and functionally (if the function of a given isoform was characterised at all) from the canonical isoform yet still may or may not harbour the oncogenic hotspot mutation.

## Alternative splicing pattern of NRAS

**Fig. 2:**
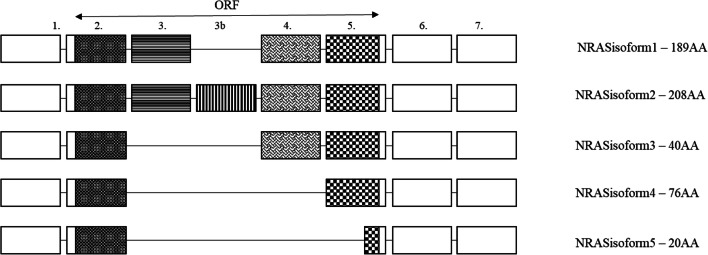
Alternative splice variants of NRAS [58]. These are the isoforms described in the literature. Ensembl only records isoform 1

Both the NCBI (NM_002524.5) and Ensembl (ENST00000369535.5) databases list only the canonical isoform (isoform 1) of NRAS in a searchable format. However, since Eisfeld et al. [58] have published their work in 2014, a number of articles have been published describing the structure, function and tissue/tumour-specific expression of four new NRAS isoforms in detail. Taking the canonical isoform 1 with its 189 amino acids and 7 expressed exons (exons 2, 3, 4 and 5 being coding) as a base, isoform 2 also contains exon 3b via *in frame* retention resulting in a 208 amino acid long protein. Δ3 exon skipping results in isoform 3. As a STOP codon is generated this way within the reading frame of exon 4, the final protein is only 40 amino acid long. Isoform 4 encodes 76 amino acids as a result of Δ3, 4 exon skipping. The coding sequence of the shortest, 20 amino acid long isoform 5 consists the first 17 codons of exon 2 and last 3 codons of exon 5 (Fig. 2). Functional studies under experimental conditions examining the effect of overexpression of isoforms 3 and 4 on the downstream targets MEK, ERK and AKT phosphorylated by isoform 1 showed lower activity of MEK and ERK and a level of activity comparable with isoform 1 of AKT (NRAS G12D was used as control). Unexpectedly, isoform 5 increased the activity of all downstream targets. Isoform 2 caused less activity along the MEK/ERK axis and increased activity of AKT. Stable transfection of the isoforms into fibroblasts showed the ectopic expression of isoform 5 to significantly increase while isoform 3 to decrease the proliferative activity of the cells. They also showed that all isoforms were present in the examined four different normal tissue types (lung, colon, skin, thyroid) as well as the tumours arising from these tissues (NSCLC, CRC, MM, PTC) within the same subject. The level of expression was different between the normal and matched tumoural tissue with the exception of lung tissue. All isoforms were identified within the cytoplasm, and isoforms 3 and 5 were also present in the nucleus. Markowitz et al. [59] showed that regardless of their BRAF mutational status, the expression level of NRAS isoform 5 is increased in human melanoma compared with normal melanocytes, which they suggested would result in an aggressive phenotype. This 20 amino acid long molecule does not contain a GTP-binding region, therefore, cannot and did not show GTPase activity similar to isoform 1. They were therefore examining the structural properties to explain the increased downstream phosphorylation of its targets (MEK, AKT, ERK) and the resulting increased proliferative activity. NMR and CD spectroscopy examinations proved that in aqueous solutions, the protein is highly flexible with no stable secondary of tertiary structure. However, in the presence of trifluoroethanol (a molecule imitating target when the actual target is unknown), it forms a helix-turn-coil structure, which might lead to better understanding of its mechanism of action via biological analogues. Duggan et al. [60] have proved in their experimental setup that the overexpression of NRAS isoform 2 increases the proliferative activity of BRAF V600E mutant human melanoma and confers resistance to BRAF inhibitor therapy. According to their results, the increased PI3K activity within the isoform 2 expressing cells is the underlying mechanism for the resistance. Follow-up RNAseq data from 423 human cutaneous melanoma by Duggan et al. [61] showed expression of all five NRAS isoforms in both the primary tumour and their metastases. Within the 23 primary and metastatic tumoural samples from a single patient with malignant melanoma, they found a positive correlation with the expression of isoform 5 across brain, liver and lung metastases as well. Their experiments using the A375 cell line showed that the overexpression of isoforms 2 and 5 correlated with *in vitro* vemurafenib resistance. Yan J et al. [62] demonstrated the high level of NRAS isoforms 3 and 5 expression to be an adverse prognostic indicator in their cohort of 140 melanoma patients.

## Alternative splicing of HRAS

**Fig. 3:**
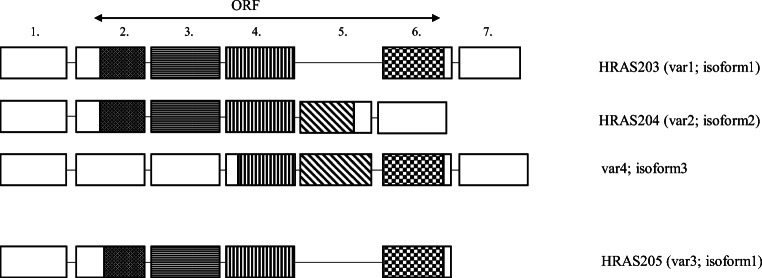
Alternative splice variants of HRAS [64]. Even today, HRAS204 is mostly referred to as p19H-RasIDX

There are four alternative splice variants of HRAS listed in both the NCBI and Ensembl databases. Variants 1 (Ensembl HRAS-203, NCBI refseq NM_005343.4, NP_005334) and 3 (Ensembl HRAS-205, NCBI refseq NM_001130442, NP_001123914) are coding the same 189AA long protein isoform (isoform 1). Their mRNA differs in the length of the 5’ and 3’ non-coding regions. Isoform 1 is considered to be the canonical, p21 protein with resulting classical HRAS functions. Compared with variant 1, transcript variant 2 of HRAS (Ensembl HRAS-204, NCBI refseq NM_176795, NP_ 789,765) carries a retained intron between exons 4 and 5. This variant with the additional alternative exon was first identified in 1989 by Cohen JB et al. [63], and they suspected its function to be the inhibition of p21 expression. A point mutation within introns 4–5 prevented the expression of the alternative splice variant coding the unknown p19 protein, which resulted in increased expression and in parallel transforming activity of p21. They found the molecule to be evolutionally conserved with only a single amino acid difference between the human and rat variants. Guil et al. [64] named the unknown alternative exon IDX in 2003. Following the nomenclature of the canonical p21H-Ras4A, they named the new molecule p19H-RasIDX (Fig. 3). The inclusion of the alternative exon is regulated by a complex process. It is negatively regulated by an intronic silencer sequence (rasISS1) positioned downstream. The inhibition is in part mediated by hnRNP A1, while inclusion is subserved by SC35 and SRp40 SR [65]. The p68 RNA helicase (DDX5) also plays a significant role in the complex process resulting in the appearance of the alternative exon. It prevents IDX inclusion by inhibiting hnRNP H binding to IDX-rasISS1. The protein coded by the alternative variant differs from the canonical p21 variant in both its biochemical and functional properties. The shorter sequence is the result of an *in frame* STOP codon within the IDX alternative exon. They also showed that the p19 protein localised in both the cytoplasm and the nucleus. It is present in a number of cell lines and human tissues in similar quantity to p21. Unlike p21, it does not bind GTP, but it binds PKCβII and SRC activating RACK1 scaffolding proteins. Jeong et al. [66] proved that it increases the expression of p73β via binding to MDM2, which would repress p73 transcription. It appears that the focus of almost all relevant publications is to stress the functional difference between the p21 and p19 proteins, including the perinuclear localisation of p19 and the fact that it does not bind the characteristic effectors (Raf1, MAXP1, AF6, and Ral-GDS) nor activators (SOS, CDC25 and p120GAP) of p21 [67]. p19 does however activate ERK1, therefore, regulating the G1/S delay by increasing the length of G1 phase. Additionally, it also induces FOXO1, hyperphosphorylates p70SK6 and Akt, therefore, preventing programmed cell death (apoptosis).

## Alternative splicing of KRAS

**Fig. 4:**
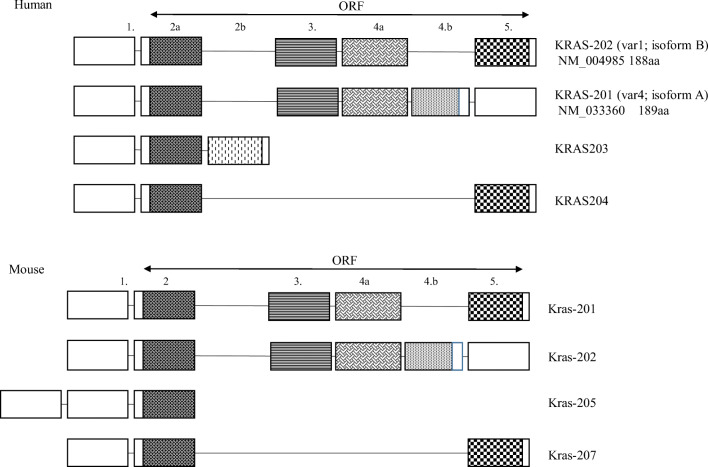
Alterative splice variants of KRAS in human (KRAS) and mouse (Kras) hosts according to the Ensembl database. Phylogenetic conservatism is evident. Interestingly, even though the introns are dramatically different between the two species, the splice sites are clearly marking identical exons

The Ensembl database lists four splice variants of human KRAS. According to data from the The Human Protein Atlas, KRAS-201 (traditionally called KRAS4A) codes a 189aa long (21.7 kDa), KRAS-202 or KRAS4B a 188aa long (21.4 kDa), KRAS-203 a 43aa long (4.7 kDa) and KRAS-204 a 75aa long (8.5 kDa) protein. Their phylogenetic conservativism is well demonstrated by the fact that the mouse equivalent of KRAS-201 (Kras-202) only differs in one amino acid from its human counterpart, while mouse equivalents of KRAS-202 (vs Kras-201) and KRAS-204 (vs Kras-207) differ in 4-4 amino acids, respectively (Fig. 4). KRAS4A and KRAS4B demonstrate well how the structural changes of alternative splice variants result in significantly different protein functions, insomuch as some publications even list KRAS4A and KRAS4B as separate standalone RAS isoforms alongside NRAS and HRAS. The number of amino acids and molecular weight of KRAS4A and KRAS4B are almost identical. However, the last 24 and 25 amino acids on their C-terminal regions are coded by different exons and are therefore significantly different, while the first 164 amino acids are identical. The different C-terminal results in the aforementioned difference in post-translational modification, namely, the palmitoylation of KRAS4a but not KRAS4B. KRAS4B is considered to be the predominant isoform even though KRAS4A is more similar to the original retroviral KRAS. There is experimental proof that similar to NRAS and HRAS, the expression of KRAS4A is essential for successful ontogenesis. The development, quality of life and lifespan of KRAS4A knockout animals are identical to those of their wild-type counterparts [68]. On the contrary, KRAS knockout mice die between days 12–14 of embryogenesis [37, 39].

Although a significant proportion of KRAS expression (60–99%) during ontogenesis is contributed by KRAS4B, its constitutive expression is in stark contrast to the organ (liver, kidney, lung, intestine) and time-specific dynamics of KRAS4A [69, 70]. The expression of KRAS4A in the last stages of mouse ontogenesis (E11.5) increases by 10–25-fold in stomach and intestine, approaching the level of KRAS4B expression. Interestingly the tissue pattern observed in embryonal tissues is mostly preserved in the fully developed animals as well [69].

According the work of Nussinov R et al. [71], functional NRAS is Janus-faced. In its palmitoylated state, it functions analogously to NRAS, while in depalmitoylated state, it behaves KRAS4B-like. Due to the reversibility of palmitoylation, KRAS4A and NRAS exist in bimodal signalling states which may take place under different oncogenic cell/tissue conditions. The structural differences of the two variants lead to different subcellular localisation and biological functions. Zhang X et al. [72] examined the nucleotide-dependent interactom network of KRAS4A and KRAS4B, constitutively active due to underlying G12D mutation in HEK293T cultures using mass spectrometry (MS)-based quantitative proteomics based on stable isotope labelling by amino acids (SILAC). Wild-type KRAS can be found predominantly (about 93%) in GDP-binding form in cells, while most base combinations of G12 mutants, with the exception of prolin, are in GTP-binding form. Despite this, all isoforms were sharing over 50% of the interacting proteins, which suggests that these are independent of the molecules' GDP/GTP status. However, when comparing the various isoforms (KRAS4A vs KRAS4B or KRAS4A G12D vs KRas4B G12D), the other 50% of interacting proteins were found to be isoform-specific. There were numerous shared biological processes, such as DNA repair, nucleotide-binding and alternative splicing. KRAS4A-specific interacting proteins are involved in mitosis, DNA damage and ion transport. KRAS4B-specific interacting proteins are involved in neurodegeneration, mRNA transport, lipid metabolism and protein biosynthesis. There were proteins interacting with only one KRAS isoform, such as v-ATPase a2 and eIF2B only interacting with KRAS4B. The localisation of v-ATPase is known to be lysosomal surface membrane bound; therefore, lysosomal localisation of KRAS4B is different to that of KRAS4A. KRAS4B may regulate protein translation initiation by interacting with eIF2B.

In contrast to the results of the Voice research group, Zhang et al. found higher affinity of Raf1 to KRAS4A as compared with KRAS4B, and as a consequence, at similar level of expression, KRAS4A G12D cells showed a higher level of ERK phosphorylation, a sign of RAF-MEK-ERK signalling activated by KRAS4A-RAF1 binding. This may explain that KRAS4A- and KRAS4B-transformed NIH 3T3 cells showed a similar proliferation in 2D, while KRAS4A-transformed cells formed a significantly higher number of colonies in soft agar assay. The biological effect was therefore found to be similar by both groups [57, 72]. As both the RAF-MEK-ERK and the PI3K-Akt-mTOR pathways play a significant role in the regulation of anchorage-independent cell growth and the latter is of similar activity in the case of both variants, they suggested that the cause of the phenomenon is increased KRas4a-RAF1 interaction and activation of the RAF1-MEK-ERK signalling cascade.

Active KRAS signalling occurs at the plasma membrane. However, the two KRAS variants can also show alternative localisation, different from each other. Interaction with the lysosome membrane-bound v-ATPase a2 links KRAS4B to the lysosome membrane, while the depalmitoylated KRAS4A is co-localised with hexokinase 1(HK1) at the outer mitochondrial membrane (OMM) with a direct GTP-dependent interaction [73]. Most of our knowledge about a number of biological functions of the two variants come from the examination of exon 4 deleted K-ras(tmDelta4A/tmDelta4A) mice, its heterozygous variant (K-ras(tmDelta4A/+) and the *in vitro* cell cultures created from them. Examining the biological effects of the two variants in the embryonic stem cells (ES) originating from these animals, authors found that the wild-type KRAS4B showed anti-apoptotic while KRAS4A showed pro-apoptotic activity [74]. It was proven in experimental carcinogenesis model (1,2-dimethylhydrazine-indukált colonic adenomas) that both homozygous and heterozygous KRAS4A knockout animals produced larger colonic adenocarcinomas with shorter survivor, i.e. wild-type KRAS4A has a tumour suppressive effect. Adenomas not expressing KRAS4A had significantly increased cell proliferation and significantly decreased apoptotic activity with evidence of activation of MAPK and Akt pathways as compared with heterozygous KO and wild-type KRAS harbouring-induced tumours [75]. Similar results were found in clinical setting, when ‘matched’ tumour and tumour-free colon tissues from the same patient showed significantly decreased KRAS4A/4B ratio in tumours harbouring both mutant and wild-type KRAS [76, 77]. Although it is considered to be a minor splice variant [76], its expression in colorectal tumours approaches and often exceeds that of KRAS4B. The alternative splicing of KRAS was found to have prognostic significance when examining mismatch stabile (MSS) colorectal cancer cases [77]. In cases of KRAS wild-type MSS colorectal cancer (stage I–III), low-level KRAS4A expression resulted in significant increase in overall survival. Such increase was not observed in cases with mutant KRAS. Accordingly, KRAS4A appeared to be an independent prognostic indicator in the wild type KRAS expressing MSS colorectal cancer.

It is less clear which factors regulate the spatial and temporal expression pattern of KRAS alternative splice variants. According to Riffo-Campos ÁL et al. [78], the change in 4A/4B ratio is most likely to be regulated by epigenetically modified histones (H3K4me3, H3K27me3, H3K36me3, H3K9ac, H3K27ac and H4K20me1). Different splice mechanisms for KRAS4A and KRAS4B seem to be operational based on the experimental results according to which inhibition of the RBM39 complex (indisulam, CRISPR/Cas) inhibits the splicing of KRAS4A exclusively with no effect on KRAS4B [79].

## Summary

The expectation that alternative splice variants of RAS, which are structurally different to the major isoform, would fulfil different functions in the life of the cell/tissue/organism has now been underpinned by published literature. As all variants described above include codons 12 and 13, therapies targeting these codons will affect not only the predominant isoform but also the alternative variants as well. Therefore, this fact can only be disregarded after careful examination and consideration of its consequences.
